# Discovery of Trypanosomatid Parasites in Globally Distributed *Drosophila* Species

**DOI:** 10.1371/journal.pone.0061937

**Published:** 2013-04-29

**Authors:** James Angus Chandler, Pamela M. James

**Affiliations:** Department of Evolution and Ecology and Center for Population Biology, University of California Davis, Davis, California, United States of America; Universidade Federal de Minas Gerais, Brazil

## Abstract

Microbial parasites of animals include bacteria, viruses, and various unicellular eukaryotes. Because of the difficulty in studying these microorganisms in both humans and disease vectors, laboratory models are commonly used for experimental analysis of host-parasite interactions. *Drosophila* is one such model that has made significant contributions to our knowledge of bacterial, fungal, and viral infections. Despite this, less is known about other potential parasites associated with natural *Drosophila* populations. Here, we surveyed sixteen *Drosophila* populations comprising thirteen species from four continents and Hawaii and found that they are associated with an extensive diversity of trypanosomatids (Euglenozoa, Kinetoplastea). Phylogenetic analysis finds that *Drosophila*-associated trypanosomatids are closely related to taxa that are responsible for various types of leishmaniases and more distantly related to the taxa responsible for human African trypanosomiasis and Chagas disease. We suggest that *Drosophila* may provide a powerful system for studying the interactions between trypanosomatids and their hosts.

## Introduction

A century of basic research in *Drosophila* genetics, physiology, ecology, and evolution has solidified its status as a model organism for biological research. Work in *Drosophila* informs applied research across a variety of disciplines, including drug discovery [1], the genetic basis of human diseases [2], [3], and the genomics of insect resistance to pesticides [4]. One area where *Drosophila* has made a particularly strong impact is the study of the animal response to microbial pathogens [5]. For example, the discovery that the intracellular bacterium *Wolbachia* reduces viral growth in *Drosophila melanogaster* [6] raises the possibility that *Wolbachia*-infected *Aedes aegypti* mosquitoes will be an effective control against dengue virus transmission [7], [8]. *Drosophila* has also proven to be a valuable model for human diseases. Cystic fibrosis (CF) in humans is commonly associated with infection by the opportunistic pathogen *Pseudomonas aeruginosa*. *Drosophila* has been developed as a model for CF and it was found that other bacterial taxa isolated from CF patients can modify *P. aeruginosa’s* role in CF afflicted *Drosophila* [9]. This polymicrobial view of CF infection is now being applied to human patients [10], [11]. Thus, the utility of using *Drosophila* as a model for host-microbe interactions is well established. However, one area where it has rarely been applied is the study of animal-trypanosomatid interactions.

Trypanosomatids (Euglenozoa, Kinetoplastea) are unicellular eukaryotic parasites of invertebrates, vertebrates, and plants [12]. Parasitic trypanosomatids can be primarily restricted to one host (monoxenous) or cycle between two hosts (dixenous). Several dixenous trypanosomatids are clinically important human pathogens that are vectored by insects. Among these are *Trypanosoma brucei*, *Trypanosoma cruzi* and various species of *Leishmania*, which are the causative agents of human African trypanosomiasis, Chagas disease, and the leishmaniases, respectively. These three neglected tropical diseases account for over 60,000 human deaths per year [13].

Monoxenous trypanosomatids have been detected in Diptera, Hemiptera, Hymenoptera, Lepidoptera, and Siphonaptera [14], [15] although the true diversity of insect-associated trypanosomatids is likely far from realized [14], [16]. Indeed, extensive surveys within Heteroptera have found that nearly a quarter of all individuals are infected, many with previously undescribed trypanosomatid strains [17], [18], [19], [20]. These surveys challenge the “one host – one parasite” view of trypanosomatid infection [21] and suggest that the factors defining host-parasite specificity are not well understood in monoxenous trypanosomatids. While the negative effects, if any, of most trypanosomatids are unknown, a common parasite of bumble bees, *Crithidia bombi*, imposes dramatic fitness consequences on hibernating queens, which subsequently leads to reduced colony-founding success [22].

Despite an abundance of studies on *Drosophila* interactions with bacteria, fungi, and viruses [5], *Drosophila*-trypanosomatid interactions have been neglected. Trypanosomatids were first found in *Drosophila confusa* [23] and were subsequently found to be prevalent in natural fly populations in both Europe and the United States [15], [24], [25]. Infections spread quickly through laboratory populations and then are maintained over the course of at least 250 days [26]. Trypanosomatids are observed in the intestines and the Malpighian tubules of flies and in laboratory media and bananas that have been used by infected individuals [15], [26]. To our knowledge, only one study has attempted to define the molecular basis of immune response to trypanosomatid infection in *Drosophila* [27]. The authors find that host survival and antimicrobial peptide production is dependent upon both trypanosomatid species and whether the parasite is ingested orally or injected directly into hemolymph.

Because *Drosophila* is an established model organism for studying host-microbe interactions and because trypanosomatids are important parasites of both humans and insects, we sought to characterize the diversity of trypanosomatids associated with *Drosophila*. Over 3000 species of *Drosophila* and related genera inhabit every continent except Antarctica, and these taxa utilize a great variety of substrates as feeding and breeding sites [28], [29]. In this study, we wanted to survey a wide breadth of host phylogenetic, geographic, and ecological diversity in order to capture the extent of trypanosomatid diversity associated with natural populations of *Drosophila.* Fourteen different species of flies from four continents were collected, with special emphasis placed on flies obtained from a variety of feeding sites, including fruits, flowers, cacti, and mushrooms. We show that *Drosophila*-associated trypanosomatids are closely related to other monoxenous insect trypanosomatids and follow the same patterns of host-infectivity and geographic distribution. We end with a discussion of how research into *Drosophila*-trypanosomatid interactions can inform trypanosomatid work in general and suggest that *Drosophila* represents a powerful and underused model for studying the transmission and virulence of these often neglected parasites.

## Methods

### Fly Collection, Dissection, and DNA Extraction

*Drosophila* samples were collected with the help of many colleagues around the world (see Acknowledgments, Table 1, Dataset S1). No specific permits were required for the described field studies and owners of private residences provided informed consent before collections took place. All samples were obtained from naturally occurring substrates, and no artificial baits were used to attract flies. For collections done in Northern California, adults were immediately transferred to sterile no-nutrient media (2% agar in water) and transported to the University of California, Davis for dissection. For more remote field collections, flies were stored in 100% ethanol for transport. For freshly collected flies, the entire gut was dissected. However, for flies stored in ethanol, dissection was not feasible because weakening of the fly tissues caused the gut to fragment. For these samples, the entire fly bodies were externally sterilized before DNA extraction. Specifically, the entire fly bodies were washed twice in 1 ml 2.5% sodium hypochlorite and twice in 1 ml sterile water with each wash consisting of 30 seconds of vortexing at max speed with 0.5 ml of 0.1 mm glass beads. Seven to 20 fly bodies or guts were combined for each sample. The detailed DNA extraction protocol can be found in [30]. Further details regarding sample collection dates, locations, and contents can be found in Dataset S1.

**Table 1:** *Drosophila* populations associated with trypanosomatids.

LibraryName	Number oftrypanosomatidSequences	Species	Diet	Location
ANM	36	*D. ananassae*	*Morinda* fruit	Captain Cook, Hawaii
ELA	65	*D. elegans*	*Alpinia* flowers	Hsinchu, Taiwan
ELD	3	*D. elegans*	*Brugmansia* flowers	Hsinchu, Taiwan
FNS	18	*D. falleni*	*Russula* mushrooms	Stony Brook, NY
ICF	55	*D. immigrans*	Citrus fruit	Wolfskill Experimental Orchard, Winters, Ca
IMH	127	*D. sp. aff. immigrans*	*Hibiscus* flowers	Captain Cook, Hawaii
MEC	46	*D. malerkotliana*	*Terminalia* fruit	Seychelles islands, Africa
MIC	8	*Microdrosophila sp.*	Shelf fungus	Malaysia
MOV	9	*D. mojavensis* and *D. arizonae*	Agria cactus	Sonora, Mexico
NNS	3	*D. neotestacea*	*Russula* mushrooms	Stony Brook, NY
POM	236	Unidentified *Drosophila sp.*	*Ipomoea* flowers	Waimanu, Hawaii
PON	5	Unidentified *Drosophila sp.*	*Pandanus* fruit	Waimanu, Hawaii
SCA	3	*Scaptodrosophila hibiscii*	*Hibiscus* flowers	Queensland, Australia
SPP	1	*D. melanogaster* and *D. simulans*	*Opuntia* fruit	Arboretum, Davis, Ca
TKM	290	*D. takahashii*	*Morinda* fruit	Captain Cook, Hawaii

Further details provided in Dataset S1.

### Library Creation and Sequencing

Details of primer design, PCR conditions, sequencing, and quality checking parameters are provided in [31]. Briefly, the D1/D2 loop of the rDNA 28S large subunit (LSU) was amplified using the primers NL1 and NL4 [32] (Dataset S2). The amplified LSU from each sample was sequenced on a Roche GS Junior Titanium machine in the laboratory of Dr. Jonathan Eisen with the assistance of the University of California, Davis Microarray Core Facility. 12819 total reads were generated. The raw sequencing reads were checked using the QIIME platform [33] resulting in 4877 high-quality reads for analysis. This dataset was previously used to describe the yeast communities associated with these same *Drosophila* populations [31].

### Initial Identification of Trypanosomatid Reads and Sequence Alignment

Initial taxonomy assignment was performed by querying each of the 4877 sequences to the entire NCBI database (as of 10/21/2011). 961 sequences had a closest match to either *Leishmania donovani* (911 sequences) or *Crithidia fasciculata* (50 sequences). These sequences will be the focus of this study. The remaining sequences have a nearest blast hit to fungi, *Drosophila,* or plants [31].

To understand why so many trypanosomatid sequences were amplified with primers primarily used for fungal identification, the NCBI primer blast tool was used to determine the specificity of the NL1 and NL4 primers. Both the primers were exactly complementary to their proposed binding sites in many fungi including common *Drosophila* associates such as species of *Hanseniaspora* and *Saccharomyces* [31] (data not shown). When these primers were queried against the genomes of the trypanosomatids, *Leishmania major*, *Trypanosoma brucei* and *Crithidia fasciculata*, it was found that the NL4 primer is exactly complementary to its proposed binding sites, whereas three mismatches occur with the NL1 primer (data not shown).

The 961 putative trypanosomatid sequences have an average length of 488 base pairs (min = 469, max = 561). 24 sequences were assumed to be chimeric and removed because an NCBI Blast search found that the 100 final base pairs were not closely related to any trypanosomatid. The remaining 937 sequences were aligned using *maffT* and the genafpair option to produce an alignment of 673 columns [34], [35]. Since many positions toward the end of the alignment (roughly corresponding to the D2 region) contained mostly gaps, the final 163 columns were removed. Despite removing these 163 columns from the alignment, an average of only 67 nucleotides was removed from each read (min = 41, max = 88).

25 sequences were identified as chimeric using the UCHIME chimera checker in *mothur* [36]. The remaining 912 sequences were re-aligned using *maffT* producing an alignment of 502 positions. Seven additional sequences were identified as chimeric in this alignment. After their removal, *maffT* was used to produce the final alignment of 905 sequences and 500 columns. The final dataset consists of 15 libraries with an average of 60 sequences each (min = 1, max = 284). Seven of the libraries contain ten or fewer sequences. The 905 sequences used in the final analysis are available through NCBI under the accession numbers KC182802 to KC183706. All sequences and alignments are available through figshare (http://dx.doi.org/10.6084/m9.figshare.106978).

### OTU Generation, Diversity Measurements and Phylogenetic Analysis

Prior to community and phylogenetic analyses, similar sequences were grouped into operational taxonomic units (OTUs). This is done because each sequence represents a different individual within the microbial community and similar sequences come from closely related individuals. OTUs are therefore surrogates for the different microbial taxa within the community. The software package *mothur* was used to generate OTUs from the chimera-checked alignment [36]. OTUs were formed at the 3% divergence level (97% similarity) using the average neighbor clustering algorithm and the countends = F option during the calculation of the distance matrix. This is the same similarity threshold used for the bacterial [30] and yeast [31] communities associated with these *Drosophila* populations. OTU clustering produced 17 OTUs with an average size of 52.2 sequences (SD = 91.3). The largest OTU contains 314 sequences, nine OTUs contain only one sequence, and 11 OTUs contain 10 or fewer sequences (Table 2, Dataset S3). OTUs were also clustered at the “unique” cutoff (identical sequences are grouped together; 0% divergence). This produced 414 OTUs with an average size of 2.2 sequences (SD = 7.1). The largest OTU contains 92 sequences, 365 OTUs contain only one sequence, and 405 OTUs contain 10 or fewer sequences (Table 3, Dataset S4).

**Table 2:** Distribution of trypanosomatids within and between *Drosophila* populations at the 3% divergence level (97% similarity).

	Sympatric					Sympatric		Sympatric		Sympatric						
Number of Sequences in OTU	ANM	IMH	POM	PON	TKM	ELA	ELD	FNS	NNS	ICF	SPP	MEC	MIC	MOV	SCA	
314	32		1		262					17		2				Both
182			180		1						1					Both
159	3		16		22	64	2	6	1			42		1	2	Both
144		127	1	2	3					10		1				Both
47			26					12					8		1	Allopatric
38									2	28				8		Allopatric

Sympatic populations were collected within the same geographic area during the same time period. All other population combinations are considered allopatric. Final column indicates if the OTU was found in either sympatric populations, allopatric populations, or both allopatric and sympatric populations. Only OTUs that are present in multiple populations are shown. Data for remaining OTUs can be found in Dataset S3.

**Table 3:** Distribution of trypanosomatids within and between *Drosophila* populations at the 0% divergence level (100% similarity).

	Sympatric					Sympatric		Sympatric		Sympatric						
Number ofSequences in OTU	ANM	IMH	POM	PON	TKM	ELA	ELD	FNS	NNS	ICF	SPP	MIC	MEC	MOV	SCA	
92	13				78								1			Both
58		56		1									1			Both
55	8				47											Sympatric
41					1	24	1						15			Both
31	3				28											Sympatric
9						6							3			Allopatric
8						4		1					3			Allopatric
6									1	5						Allopatric
3									1	2						Allopatric
2						1							1			Allopatric
2	1				1											Sympatric
2	1				1											Sympatric

Sympatic populations were collected within the same geographic area during the same time period. All other population combinations are considered allopatric. Final column indicates if the OTU was found in either sympatric populations, allopatric populations, or both allopatric and sympatric populations. Only OTUs that are present in multiple populations are shown. Data for remaining OTUs can be found in Dataset S4.

A representative sequence for each 3% divergence OTU was chosen using the get.oturep function in *mothur* which selects the sequence that has the minimum total distance to all the other sequences within that OTU. Kinetoplastid LSU sequences were taken from NCBI for phylogenetic comparison (Dataset S5). These include several species of monoxenous insect trypanosomatids (*Leptomonas*, *Herpetomonas,* and *Crithidia*) and numerous dixenous insect-vectored trypanosomatids (*Leishmania*, *Endotrypanum*, and various *Trypanosoma* species). The free living outgroup to the order trypanosomatida (*Bodo saltans* of the order Bodonida) was used as the outgroup in this analysis [12]. These Kinetoplastid sequences were aligned to the degapped, aligned representative sequences using maffT, the genafpair algorithm, and the add function. Because several of these sequences are very long (over 10,000 bases) and span the entire ribosomal repeat region (which includes the 5S, 18S, and 28S rDNA genes and the ITS1 and ITS2 interspacer regions), this alignment was trimmed to include only the 28S rDNA (LSU) region. jModelTest was used on this trimmed alignment to determine the optimal model of nucleotide substitution (GTR+G) [37], [38]. Bayesian analysis was performed using MrBayes v3.1.2 [39]. Two independent chains were run for 10,000,000 generations resulting in an average standard deviation of split frequencies of 0.0040. Tracer v1.5.0 was used to confirm stationarity of the log likelihoods [40], and the first 25% of the 10,000 total trees were used as burnin. Results were visualized using Dendroscope [41].

As many of the libraries have very few sequences, diversity analyses will be limited to interpretations that can be determined by shared presence. Alpha and beta diversity measurements and *UniFrac* analysis [42] were not performed.

### Characterization of the SSU in a Single Population of *Drosophila*

For 14 of the 15 populations in which trypanosomatids were discovered, no whole flies or DNA remained. However, for one population (*Drosophila ananassae* collected in Hawaii; ANM in Table 1), numerous flies remained from the initial collection and could be used to study between individual and within individual variation in trypanosomatid infection. Additionally, a different diagnostic gene, the 18S rDNA small subunit (SSU), can be used to refine the phylogenetic placement of the *Drosophila*-associated trypanosomatids. The SSU is a commonly used diagnostic marker for trypanosomatid identification [43].

DNA was extracted from 21 individual flies. Single flies were homogenized in 250 ul of HB buffer (50 mM Tris, 400 ml NaCL, 20 mM EDTA, 0.5% SDS, pH 7.5) followed by a three hour 55C Proteinase K incubation and ethanol precipitation. Successful DNA extraction was confirmed by PCR amplification using the *Drosophila* specific primers COII-F and COII-R (Dataset S2). Any samples that did not amplify were not used in further analyses. The amplified COII gene was sequenced to confirm host identity and is available through NCBI under the accession number KC183710.

To test flies for the presence of trypanosomatids, the primers SSU1 and SSU2 were used to amplify the trypanosomatid rDNA small subunit (SSU) (Dataset S2) [44] using the following PCR protocol: Initial denaturation at 95C for 3 min followed by 30 amplification cycles (95C for 30 s, 55C for 1 min, 72C for 2 min 30 s) and a final extension at 72C for 10 min. Six individual flies tested positive for trypanosomatid infection. From these individuals, amplified DNA was ligated into the pCRII vector using the Invitrogen TOPO TA cloning kit and transformed into chemically competent DH5-alpha cells. Fifty total colonies were picked and sequenced using Life Technologies™ Big Dye ® Terminator v3.1. Between one and 17 colonies were sequenced per individual (Table 4).

**Table 4:** Distribution of trypanosomatids within and between individual flies in Hawaiian *Drosophila ananassae.*

	Individual Fly ID					
	2	4	6	8	13	21
OTU 1		1				
OTU 2		2				
OTU 5			5			
OTU 7			6		1	
OTU 35	5	14		5		11
Total Clones from Individual	5	17	11	5	1	11

OTUs named based upon the number of sequences within that OTU.

Initially, each clone was sequenced using only the SSU1 primer. These sequences were aligned and clustered using the programs and settings outlined above. A representative sequence from each of the five 3% divergence OTUs was chosen and sequenced using internal sequencing primers to ensure near-complete coverage of the SSU gene (Dataset S2). These sequences were aligned to taxa from an existing SSU dataset [43], the bumble bee parasite *Crithidia bombi*, the human parasites *Trypanosoma cruzi* and *Trypanosoma brucei,* and to their closest NCBI blast hit (as of 10/10/2012) using maffT and the genafpair algorithm. Fifteen sequences from the subfamily Leishmaniinae were removed from [43] prior to alignment. As with the LSU phylogeny, the free living *Bodo saltans* (of the order Bodonida) was used as the outgroup [12]. jModelTest was used on this alignment to determine the optimal model of nucleotide substitution (GTR+I+G) [37], [38]. Bayesian analysis was performed using MrBayes v3.1.2 [39]. Two independent chains were run for 10,000,000 generations resulting in an average standard deviation of split frequencies of 0.0025. Tracer v1.5.0 was used to confirm stationarity of the log likelihoods [40], and the first 25% of the 10,000 total trees were used as burnin. Results were visualized using Dendroscope [41]. The nearly full-length, assembled representative sequences from each SSU OTU are available through NCBI under the accession numbers KC183711 to KC183715. All sequences and alignments are available through figshare (http://dx.doi.org/10.6084/m9.figshare.106978).

## Results

### Phylogenetic Position of *Drosophila*-associated trypanosomatids

**Figure 1:**
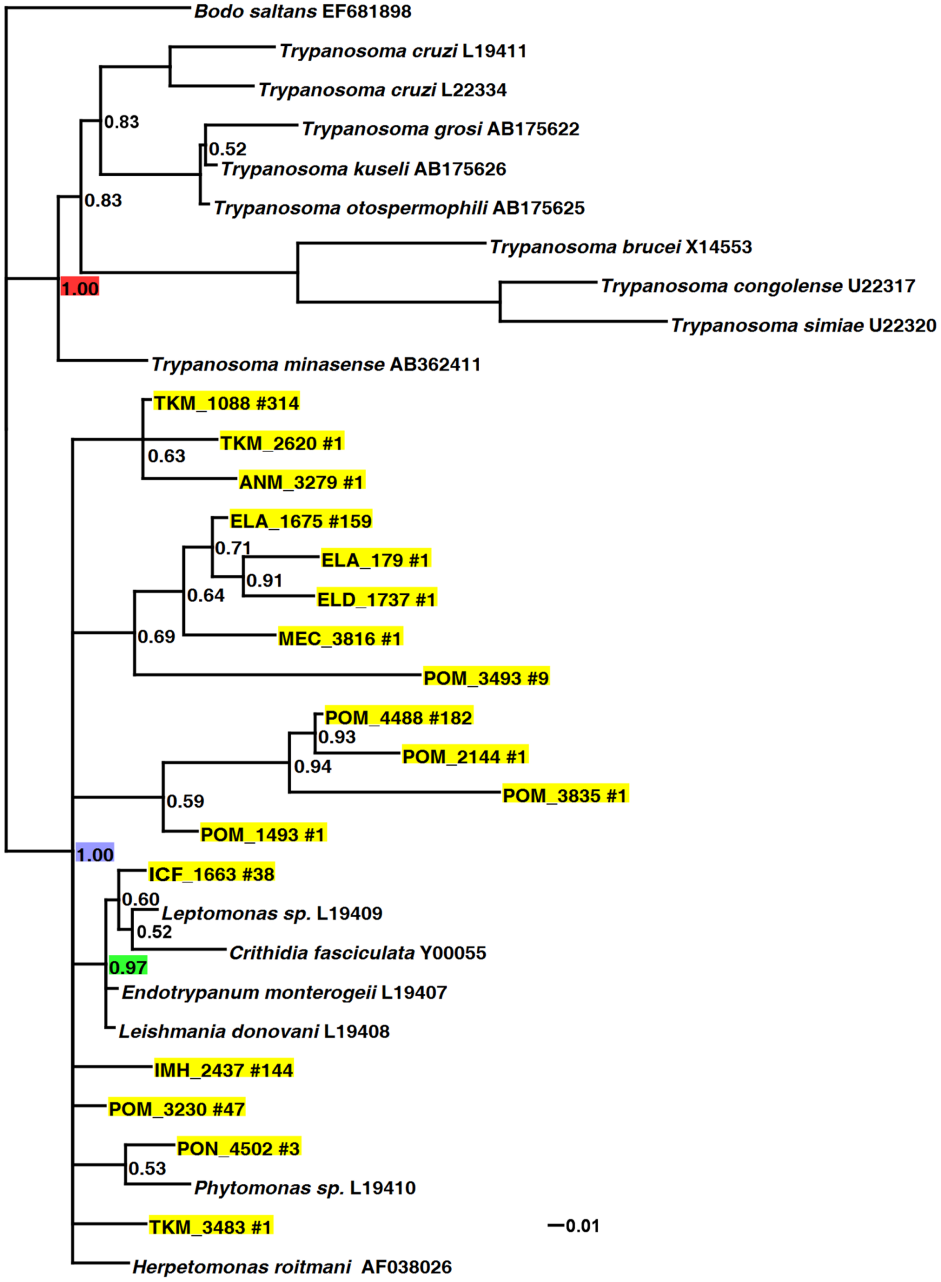
Bayesian analysis of the ribosomal large subunit (LSU) of *Drosophila* associated trypanosomatids. LSU data was obtained from 15 geographically dispersed *Drosophila* populations. Nodes with less than 50% posterior probability are collapsed. Nodes without a support value shown have 100% posterior probability. The red node identifies the genus *Trypanosoma*, the blue node identifies the non-*Trypanosoma* trypanosomatids, and the green node identifies the subfamily Leishmaniinae [45]. Representative sequences of each *Drosophila*-associated OTU are highlighted in yellow. Each representative sequence has a unique identifier followed by the number of sequences within that OTU. Unhighlighted taxa are comparison sequences obtained from NCBI and are followed by their accession number. Raw data, alignments, and the NEWICK tree file are available on figshare (http://dx.doi.org/10.6084/m9.figshare.106978).

**Figure 2:**
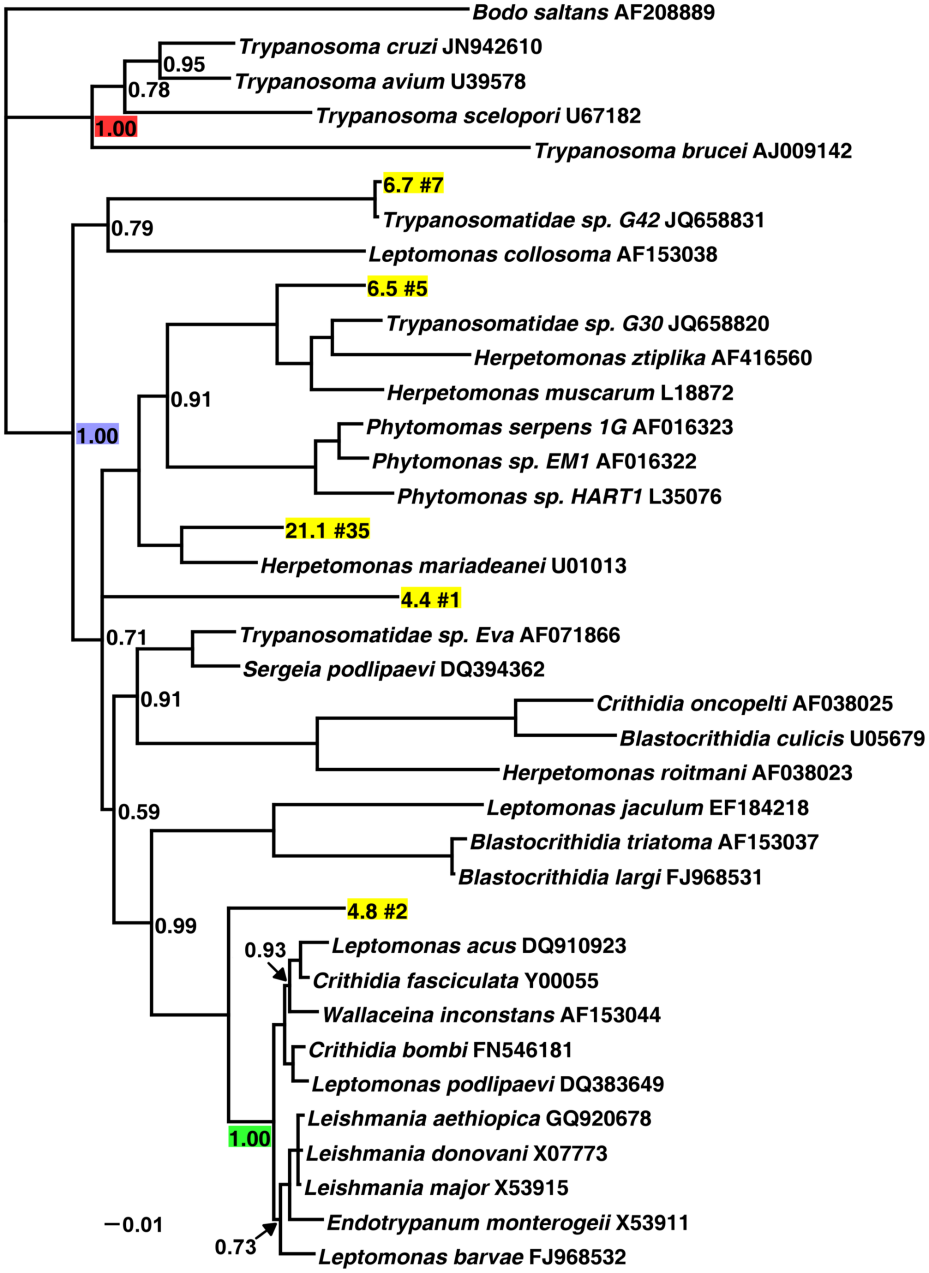
Bayesian analysis of the ribosomal small subunit (SSU) of *Drosophila ananassae*-associated trypanosomatids. SSU data was obtained from six individual flies collected in Captain Cook, Hawaii. Nodes with less than 50% posterior probability are collapsed. Nodes without a support value shown have 100% posterior probability. The red node identifies the genus *Trypanosoma*, the blue node identifies the non-*Trypanosoma* trypanosomatids, and the green node identifies the subfamily Leishmaniinae [45]. Representative sequences of each *Drosophila*-associated OTU are highlighted in yellow. Each representative sequence has a unique identifier followed by the number of sequences within that OTU. Unhighlighted taxa are comparison sequences obtained from [43] and NCBI and are followed by their accession number. The raw sequences, alignments, and the NEWICK tree file are available on figshare (http://dx.doi.org/10.6084/m9.figshare.106978).

The trypanosomatids found with *Drosophila* are closely related to other insect-associated trypanosomatids and are phylogenetically distinct from the dixenous human pathogens in the genus *Trypanosoma*. In both the SSU and the LSU phylogenetic trees, we find strong support for the clade containing the genera *Leishmania, Phytomonas, Crithidia, Herpetomonas, Endotrypanum,* and the *Drosophila*-associated sequences (Figures 1 and 2; blue node). In congruence with the current understanding of trypanosomatid phylogenetics [12], [14], both trees find strong support for the *Trypanosoma* genus (red node).

The SSU phylogeny finds that the trypanosomatids associated with the Hawaiian *Drosophila ananassae* population do not form a single monophyletic clade but belong to multiple, well supported groups (Figure 2). While the LSU phylogeny suggests this as well, many nodes in that phylogeny are weakly supported (Figure 1). The SSU data finds that no *Drosophila ananassae*-associated trypanosomatids are within the subfamily Leishmaniinae [45] (Figure 2; green node), which includes the dixenous human pathogen *Leishmania* and the mosquito parasite *Crithidia fasciculata* [44], however the LSU phylogeny finds that some of the discovered taxa (the 38 sequences represented by ICF_1663 in Figure 1) are indeed closely related to the subfamily Leishmaniinae. Several trypanosomatids found associated with *Drosophila ananassae* (the seven sequences represented by 6.7 in Figure 2) are very closely related to taxa found with Reduviidae bugs in Ghana [20].

### Trypanosomatids are Widespread and not Restricted to a Single Location

Trypanosomatids were detected in 15 *Drosophila* populations (Table 1, Dataset S3). These 15 populations come from four different continents (Africa, Australia, North America, and Asia) and seven different geographically isolated locations (Hawaii, both coasts of North America, Australia, Africa, Malaysia, and Taiwan). *Drosophila* collected from four different feeding substrates (fruit, flowers, cacti, and mushrooms) were found with trypanosomatids. At least 13 different species of *Drosophila* were associated with detectable levels of trypanosomatids.

Although there were five *Drosophila* populations with fungal sequencing reads [31] that did not have trypanosomatid reads (Dataset S1), we note that our primers were not 100% complementary to known trypanosomatid genomes (data not shown). Indeed, the number of fungal reads is inversely proportional to the number of trypanosomatid reads in a given population (data not shown) suggesting that competition for primer binding sites may be responsible for some of the apparent variation in trypanosomatid abundance between host populations.

### Closely Related Trypanosomatids are Found in Multiple *Drosophila* Populations

Some populations were collected in the same general location on the same day or, at most, within the same month. Although different host species were sampled, their physical proximity allows for the opportunity of direct cross infection between populations. These population combinations (ANM:IMH:POM:PON:TKM, FNS:NNS, ELA:ELD, and ICF:SPP) shall henceforth be called “sympatric”. All other combinations are separated by at least 1,500 kilometers and shall be called “allopatric”.

When calculated at the 3% divergence level, eight OTUs are found more than once, and the six largest OTUs (out of 17 total) are found in multiple populations. All six of these are found in allopatric populations (Table 2, Dataset S3). The most widespread OTU (comprised of 159 sequences) is found in six allopatric regions ranging from Hawaii, Australia, Taiwan, Africa, and both coasts of North America.

OTUs were also calculated at the 0% divergence level. That is, sequences had to be identical (in the aligned, trimmed dataset) to be grouped together. Although divergence at diagnostic genes may not accurately reflect divergence at genomic loci important for host adaptation, this is the most stringent cut-off available given our data. At the 0% divergence level, 49 sequences were found more than once (Table 3, Dataset S4). Of these, 12 were found in multiple populations and eight in allopatric populations. *D. takahashii* and *D. ananassae* (both collected from Morinda fruit at Captain Cook, Hawaii) share many OTUs.

### Multiple Strains within each *Drosophila* Population and within Individual Flies

Each population had more than one 3% divergence LSU OTU (Table 2, Dataset S3), and these likely represent multiple species. The population harboring the greatest diversity of trypanosomatids (POM) is associated with nine different LSU OTUs. Even though the genome of a single species may contain multiple copies of the LSU, it is unlikely that they have diverged greater than 3%. Indeed, all six copies of this region within *Leishmania major*, for which the complete genome is available, are identical (data not shown). Surveying the diversity of SSU from individual flies finds that multiple strains (at 3% divergence) can be present within a single individual (Table 4).

## Discussion

Our results provide the most extensive survey to date of trypanosomatids associated with *Drosophila* species. We find that flies from all geographic regions and feeding types are associated with trypanosomatids. Although deeper and more extensive sampling with more specific primers is needed to conclusively say if any populations or species of *Drosophila* do not harbor trypanosomatids, our data suggests that this is an unlikely possibility. This is in concordance with previous studies [24], [25], and, to our knowledge, all populations of *Drosophila* that have been explicitly tested for the presence of trypanosomatids have been infected.

The data presented here also represents the most detailed taxonomic characterization of *Drosophila*-associated trypanosomatids. Microscopy based identifications have been unable to classify taxa to below the order trypanosomatid [24]. The only study which utilized molecular methods of identification focused primarily on the hyper-variable spliced leader sequence and was therefore unable to compare *Drosophila*–associated trypanosomatids to named taxa [25]. That study also generated three sequences of the trypanosomatid GAPDH gene and found, as we did, that *Drosophila-*associated trypanosomatids are closely related to other monoxenous insect trypanosomatids.

Unfortunately, we are unable to classify many of the *Drosophila*-associated trypanosomatids discovered in the global survey (Figure 1) due to the paucity of LSU data from trypanosomatids. Indeed, most of the non-*Trypanosoma* trypanosomatids included in our analysis (*Herpetomonas roitmani, Endotrypanum monterogeii, Leishmania donovani, Phytomonas sp.,* and *Leptomonas sp.*) come from a single study [46], and, to our knowledge, no other monoxenous taxa have LSU sequence data publically available for the region overlapping the one sequenced in this study.

In addition to confirming the association of trypanosomatids with *Drosophila*, these results also provide insight into more general issues regarding the distribution, geographic endemism, and host-specificity of insect-associated trypanosomatids. The “one host – one parasite” paradigm [21] has been challenged recently by extensive surveying of the trypanosomatids associated with insects in Central America [19], China [17], and Africa [20]. Indeed, here we find that species, populations, and individuals can be associated with multiple strains of trypanosomatids. Additionally, geographically distant fly populations are associated with very closely related trypanosomatids suggesting that geographic distances do not provide a substantial barrier to dispersal relative to the evolutionary rate of the genetic marker used (LSU). A similar pattern has been observed for trypanosomatids associated with widely distributed populations of herbivorous insects in the family Pyrrhocoridae [47]. Finally, we find that *Drosophila*-associated trypanosomatids do not form a single monophyletic clade within the trypanosomatid phylogeny. This, along with the fact that sympatric species of *Drosophila* share many OTUs, suggests that little host specificity exists over both ecological and evolutionary timescales.

*Drosophila* has been used previously to study the interaction between insects and unicellular eukaryotic parasites. For example, the development of *Plasmodium gallinaceum*, a close relative of the human malarial parasite, has been modeled in *Drosophila melanogaster* by directly injecting *Plasmodium* ookinetes into the insect’s hemocoel [48]. Use of *D. melanogaster* genetic knock-outs has led to the discovery of genes in *Anopheles gambiae* that reduce *Plasmodium* growth [49]. This was possible despite the fact that *Plasmodium* does not stably infect the intestines of *D. melanogaster* [48], and no parasites of the phylum Apicomplexa, of which *Plasmodium* is a member, have ever been found in *Drosophila.* While the development of *T. brucei*, *T. cruzi,* and *Leishmania* is relatively well understood within their respective insect vectors [50], [51], [52], none of these insects can be as easily genetically manipulated as *D. melanogaster.* Because of this, we suggest that modeling trypanosomatid development in *D. melanogaster* may provide insights similar to those already gained in the *Plasmodium*-*Anopheles* system.

It is becoming increasingly clear that vertically-inherited intracellular symbionts can have a strong effect on vector infection status and disease transmissibility [53]. For example, *T. brucei* is more prevalent in tsetse flies that are co-infected with *Sodalis glossinidius* suggesting that this endosymbiont increases its host’s capacity to acquire and potentially transmit *T. brucei* [54]. In contrast, the presence of *Wolbachia* in *D. melanogaster* protects against viral infection [6], and this information is currently being used to interrupt the spread of dengue virus by *Aedes aegypti* mosquitoes [7], [8]. A reduction in pathogen load seems to be a general effect of *Wolbachia* as indicated by reduced *Plasmodium* levels in *Wolbachia*-infected mosquitoes [55]. Given that the intracellular symbionts associated with *Drosophila* are well characterized [56] and the experimental tools needed for *Wolbachia* manipulation are available in *Drosophila* [57], it seems relevant to ask how intracellular symbionts may interact with trypanosomatid parasites co-occurring in the same hosts. The results of such studies could have a substantial positive effect on efforts to control trypanosomatid caused human diseases.

## Supporting Information

Dataset S1 ***Drosophila*** **populations associated with trypanosomatids (Detailed Version).** A more detailed version of Table 1 describing where, when, and by whom each sample was collected.
(XLSX)

Dataset S2 **Primer Sequences.**
(XLSX)

Dataset S3 **Detailed OTU information at the 3% divergence cutoff.** This excel file contains the OTU assigned to each sequence used in this study along with information regarding the host species, location, environment, and other information regarding the library each sequence belongs to.
(XLSX)

Dataset S4 **Detailed OTU information at the 0% divergence cutoff.** This excel file contains the OTU assigned to each sequence used in this study along with information regarding the host species, location, environment, and other information regarding the library each sequence belongs to.
(XLSX)

Dataset S5 **Accession numbers.**
(XLSX)
